# Effects of Valley Topography on Acoustic Communication in Birds: Why Do Birds Avoid Deep Valleys in Daqinggou Nature Reserve?

**DOI:** 10.3390/ani12212896

**Published:** 2022-10-22

**Authors:** Songkai Guo, Wenhui Wu, Yaxin Liu, Xiaofang Kang, Chunwang Li

**Affiliations:** 1 Key Laboratory of Animal Ecology and Conservation Biology, Institute of Zoology, Chinese Academy of Sciences, Beijing 100101, China; 2 Institute of Physical Science and Information Technology, Anhui University, Hefei 230039, China; 3 College of Life Science, Hebei University, Baoding 071002, China; 4 College of Life Science, University of Chinese Academy of Sciences, Beijing 100049, China

**Keywords:** avian, acoustic communication, habitat structure, dense forests, open woodland

## Abstract

**Simple Summary:**

Landscape structure may influence animal acoustic communication. Our playback experiments showed that the acoustic intensity and frequency of bird vocalizations differed between the upper and lower valley. Valley topography on acoustic communication could lead birds to avoid deep valleys.

**Abstract:**

To investigate the effects of valley topography on the acoustic transmission of avian vocalisations, we carried out playback experiments in Daqinggou valley, Inner Mongolia, China. During the experiments, we recorded the vocalisations of five avian species, the large-billed crow (*Corvus macrorhynchos* Wagler, 1827), common cuckoo (*Cuculus canorus* Linnaeus, 1758), Eurasian magpie (*Pica pica* Linnaeus, 1758), Eurasian tree sparrow (*Passer montanus* Linnaeus, 1758), and meadow bunting (*Emberiza cioides* Brand, 1843), at transmission distances of 30 m and 50 m in the upper and lower parts of the valley and analysed the intensity, the fundamental frequency (F0), and the first three formant frequencies (F1/F2/F3) of the sounds. We also investigated bird species diversity in the upper and lower valley. We found that: (1) at the distance of 30 m, there were significant differences in F0/F1/F2/F3 in Eurasian magpies, significant differences in F1/F2/F3 in the meadow bunting and Eurasian tree sparrow, and partially significant differences in sound frequency between the upper and lower valley in the other two species; (2) at the distance of 50 m, there were significant differences in F0/F1/F2/F3 in two avian species (large-billed crow and common cuckoo) between the upper and lower valley and partially significant differences in sound frequency between the upper and lower valley in the other three species; (2) there were significant differences in the acoustic intensities of crow, cuckoo, magpie, and bunting calls between the upper and lower valley. (3) Species number and richness were significantly higher in the upper valley than in the lower valley. We suggested that the structure of valley habitats may lead to the breakdown of acoustic signals and communication in birds to varying degrees. The effect of valley topography on acoustic communication could be one reason for animal species avoiding deep valleys.

## 1. Introduction

Acoustic signals are one of the most important information carriers and are widely used in a variety of activities such as social interactions [1,2,3]. The exchange of information among individuals via acoustic signals is usually limited by environmental factors [4]. In some habitats, that is the continuous masking of calls by high-intensity background noise. For example, fast-moving streams or waterfalls seriously affect acoustic communication [5]. Animal sound communication is also affected by other factors, such as the amplitude of the source sound, the level of ambient noise, the ability of the sound to penetrate the environment, and the auditory sensitivity of the receiver. In addition, differences in signal frequency and the nature of the transmission medium affect the propagation of signals [6,7,8,9]. These variables are commonly studied in natural habitats through playback of natural and synthetic sounds and should be carefully considered in studies on auditory communication [10].

Guibard uses models to show how mountain conditions affect the surface and shape of active spaces, with topography being the most significant factor [11]. To study communication in birds, the bioacoustic notion of active space (AS) was introduced by Marten and Marler (1977) as being the ‘effective distance’ of a signal, the distance from the source over which signal amplitude remains above the detection threshold of potential listeners [12]. Indeed, the detection of acoustic signals is influenced by the environments between the sound source and receiver [13]. Dabelsteen et al. (1993) found that the difference in degradation between low and high microphone heights explains why the blackbird (*Turdus merula*) tends to perch on low brush [14]. Compared with the birds in woodland, the birds in forests have to broadcast their songs through more vegetation and over longer distances [15]. Therefore, forest birds reduce the attenuation of their calls in vegetation-dense environments by using low-frequency sounds, which propagate well in such habitats [16].

Fundamental frequency (F0) and formant frequency are two parameters that have been comprehensively discussed in vertebrates [17,18]. According to the source–filter theory, F0 is determined by the source signal, which is generated by the vibration of vocal folds in the larynx, whereas formant frequencies are selectively amplified when the source signal passes through the vocal tract [19].

In addition, vocal intensity has been studied as an auditory distance cue [20,21] and as a response to environmental noise in different species [22]. Generally speaking, vocal intensity in the wild is very difficult to measure owing to the effects of attenuation and degradation [4]. Nevertheless, the vocal behaviours of many taxa have been studied through acoustic analysis and synthesis techniques [23,24,25].

In this study, we selected five bird species and investigated the relationship between the transmission of their vocalisations and topography in the upper and lower parts of the Daqinggou valley in Daqinggou National Nature Reserve, China. We examined the relationships between three acoustic parameters (acoustic intensity, F0, and formant frequency) and habitat type regarding topographical structure. We also investigated the bird species diversity in the upper valley and lower valley to find which habitat sustains more species of birds.

## 2. Methods

### 2.1. Study Site and Study Species

**Figure 1:**
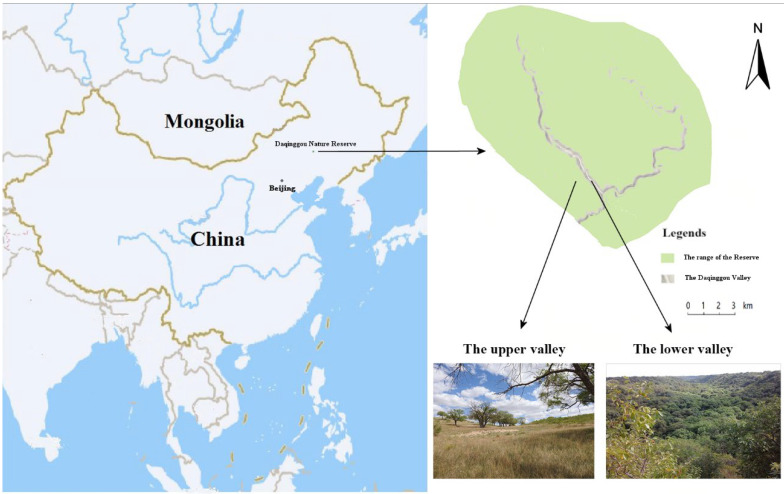
Study area and the location of the Daqinggou Valley in the Daqinggou National Nature Reserve.

This study was conducted in Daqinggou National Nature Reserve (122°13′–122°15′ E, 42°45′–42°48′ N), which is located in the southwest dune field in eastern Inner Mongolia, China. The Daqinggou valley is 200–300 m wide and 40–50 m deep, stretching over 24,000 m along an arc from east to west (Figure 1). The average annual temperature is 5.8 °C and the average annual precipitation is 0–450 mm. The upper valley was typical Horqin sandy land, and the lower valley was virgin forest. The dense forest in the lower valley was in sharp contrast to the sandy landscape in the upper valley, and the habitat conditions were very special. From the lower valley to the upper valley, although the elevation difference is only 60 or 70 m, due to the different water sources from the lower valley, the soil conditions were very different, forming different community types. The vegetation is temperate deciduous broad-leaved forest. The lower part of the valley features mainly northern Chinese flora with springs and streams scattered in the woods, whereas the upper part of the valley is formed of mainly open woodland with steppe plants. In the upper and lower parts of the valley, there were *Ulmus macrocarpa* Hance, 1868, and *Fraxinus mandshurica* Rupr., 1857, respectively [26]. We experimented with a total of five representatives of birds that were common species in Daqinggou National Nature Reserve. The five species studied were the large-billed crow (*Corvus macrorhynchos* Wagler, 1827), common cuckoo (*Cuculus canorus* Linnaeus, 1758), Eurasian magpie (*Pica pica* Linnaeus, 1758), Eurasian tree sparrow (*Passer montanus* Linnaeus, 1758), and meadow bunting (*Emberiza cioides* Brand, 1843).

### 2.2. Playback Experiments

Controlled playback experiments were performed from May to June 2014 to investigate whether acoustic communications were different between the lower and upper valley. We downloaded the bird sounds for the sound playback trials from website recordings—https://avibase.bsc-eoc.org and https://dibird.com/species/ (accessed on 12 May 2014). We used the same recordings played in both habitats of the two parts of the valley. At each randomly chosen site, we played the bird sound of each species 6 times from 7:00 to 9:00 on 23–28 June. A normal digital recorder with a directional microphone (VASO VM-398N) was used to record the sound played by a computer-connected speaker (JBL-MRX615, Beijing Haosheng, China). After reviewing all recordings, we discarded low-quality vocalisations recorded from a remote distance beyond 50 m or with high-level background noises. It was reported that signal strength decays or attenuates with increasing distance between the source and receiver [13,27]. After considering the function of vocalisations in distance and the quality of the recordings, we eventually chose 30 m and 50 m as the experimental distances. A total of 53 vocalisations were recorded at 30 m and 47 vocalisations at 50 m. Eventually, the sample size of the bird in each experimental treatment varied from three to eight. The detailed sample size was listed in the results. The recordings were transferred to a computer in WAV format with Adobe Audition 3.0 at a sampling rate of 44.1 KHz and 16-bit resolution.

### 2.3. Acoustic Analysis

Acoustic analysis of the recordings was performed by PRAAT DSP 5.3.34 [28]. We took the same part of the recordings from each species. Narrow-band spectrograms (window length = 0.04 s, time step = 0.002 s, species-dependent maximum frequency, Gaussian window shape) were plotted using the command ‘to Spectrogram’. Mean F0 was extracted using the command ‘to Pitch’ (time step = 0.01 s, Pitch floor = 75 Hz, species-dependent Pitch ceiling). The ‘Pitch Tier’ was used to check and adjust potential abnormal data. Uncertain data were identified by spectrum (slice) analysis. Then, the mean F0 and mean formant frequency of each species were determined by averaging the data of all recordings under the same conditions.

**Figure 2:**
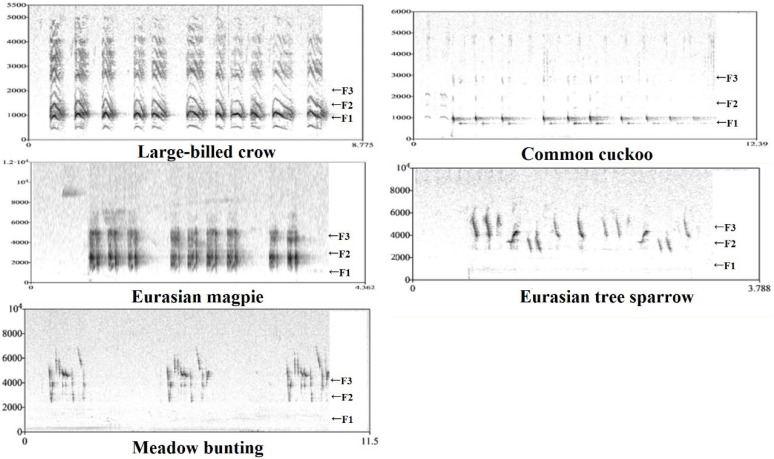
The narrow-band spectrogram of the sound of five birds extracted by PRAAT. The F1, F2 and F3 represent the first, the second and the third formant, respectively.

Formant frequency was estimated by linear predictive coding (LPC). The first three formant frequencies (F1/F2/F3) were evident on spectrograms of the sounds of five avian species (Figure 2). First, the command ‘to Formants (burg)’ (time step = 0.00625, maximum number of formants = 4, window length = 0.025 s, pre-emphasis from = 50 Hz, species-dependent maximum formant) was used to obtain the mean frequency of three formants for each recording. To ensure the accuracy of the data and to modify machine errors, we used the command ‘to LPC (autocorrelation)’. Specifically, the sound (Sound: Convert-Resample) was sampled at a rate of 11,000 Hz. Then ‘to LPC (autocorrelation)’ was run (prediction order = 10/11/16, window length = 0.025 s, time step = 0.005 s, pre-emphasis frequency = 50 Hz). Then, F1/F2/F3 were calculated using the command ‘to Spectrum (slice)’ along the recording. These data were helpful in evaluating the results of automatic formant analysis. Finally, we calculated the overall formant spacing of each recording as follows: DF = (F3 − F1)/2.

Acoustic intensity was the physical quantity that describes the intensity of sound, that is, the sound energy per unit area perpendicular to the direction of sound wave propagation per unit time. The mean vocal intensity of all recordings of each species in the lower valley (or upper valley) was determined using the command ‘to Intensity’ (minimum Pitch = 100 Hz, time step = 0.008 s). The results were checked and viewed via the ‘Down to Intensity Tier’. 

### 2.4. Line Transects to Measure Species Diversity

In May 2019, eight transect lines measuring 3000 m were randomly placed between the upper (four transect lines) and lower valley (four transect lines), with a distance between two parallel lines of at least 1 km. The species and number of birds were recorded according to the individuals and sounds encountered on each sample line.

### 2.5. Statistical Analysis

All statistical analyses were conducted in SPSS 22.0 (SPSS Inc., Chicago, IL, USA). Firstly, the distributions of all acoustic parameters and bird species diversity indices were examined via the Kolmogorov–Smirnov test, which showed that all parameters followed a normal distribution (*p* > 0.05). We used independent samples *t*-test to test the differences in the acoustic parameters and the bird species diversity indices between the upper valley and the lower valley. In the statistical procedure, we also used Levene’s test to estimate the homogeneity of variances (when *p* > 0.05, the variances were equal; otherwise, the variances were unequal). All values were presented as untransformed means ± SE. The significance level was set at *p* < 0.05 for all statistical analyses.

## 3. Results

### 3.1. Differences of Acoustic Parameters

At the distance of 30 m, there were significant differences in all the sound frequencies of the Eurasian magpie between the upper and lower valley (*p* < 0.05, Table 1). In the meadow bunting and Eurasian tree sparrow, there were significant differences in the F1/F2/F3 between the upper and lower valley (*p* < 0.05, Table 1). The large-billed crow showed significant differences in F2/F3 between the upper and lower valley (*p* < 0.05, Table 1), whereas the common cuckoo was only significantly different in F3 between the upper and lower valley (t = 5.284, df = 7.66, *p* = 0.001, Table 1).

At the distance of 50 m, there were significant differences in the F0/F1/F2/F3 of two of the birds (large-billed crow and common cuckoo) between the upper and lower valley (*p* < 0.05, Table 2). In the Eurasian magpie, no significant differences were found in any sound frequencies between the upper and lower valley (*p* > 0.05, Table 2). The Eurasian tree sparrow showed significant differences in the F1/F2/F3 between the upper and lower valley (*p* < 0.05, Table 2). The meadow bunting was significantly different in F0/F3 between the upper and lower valley (*p* < 0.05, Table 2).

### 3.2. Differences of Acoustic Intensity

At the distance of 30 m, there were significant differences in acoustic intensity between the upper and lower valley in all of the avian species (*p* < 0.05, Table 3). Meanwhile, the acoustic intensity of the calls of the meadow bunting and Eurasian tree sparrow was significantly lower in the lower valley than in the upper valley, but that of the calls of the Eurasian magpie, common cuckoo, and large-billed crow was significantly higher in the lower valley.

At the distance of 50 m, there were significant differences in acoustic intensity between the upper and lower valley in the large-billed crow, common cuckoo, Eurasian magpie, and meadow bunting (*p* < 0.05, Table 3); however, there were no significant differences in acoustic intensity between the upper and lower valley in the Eurasian tree sparrow (t = −2.041, df = 8, *p* = 0.076, Table 3). The acoustic intensity of the calls of the meadow bunting and Eurasian tree sparrow was significantly lower in the lower valley than in the upper valley, whereas that of the calls of the Eurasian magpie, common cuckoo, and large-billed crow was significantly higher in the lower valley.

### 3.3. Bird Diversity

There were significant differences in species number and richness between the upper and lower valley, with both being significantly higher in the upper valley (species number: *t* = −3.922, df = 6, *p* = 0.008; species richness: *t* = −4.084, df = 6, *p* = 0.006; Table 4).

## 4. Discussion

In terms of call properties, the fundamental and dominant frequencies of a call contribute the most to discrimination between individuals [29]. Experiments on the buff-breasted flycatcher (*Empidonax fulvifrons*) demonstrate significant distinctiveness in songs between individuals [30]. We found that the fundamental and formant frequencies of the birds studied in this experiment were significantly different in the sandy land in the upper valley and the virgin forest at the lower valley, indicating that the habitat with different topography may affect the calls of bird species.

Acoustic degradation during transmission presents a selection challenge for animals that depends on vocal communication. Environmental factors can mask communication signals, affecting the evolution of signal form and decisions about when and where to communicate [31]. Since acoustic communication can be considerably impaired by ambient noise, some animals have evolved to counteract this masking effect [32]. Not all sounds propagate equally in a given habitat; sound selection should favour the use of particular frequencies, intensities, and sound structures that carry information over the required distance, rather than the longest distance [33]. The habitat types have higher densities of physical structures that may impede or scatter sound, relative to open habitats [13]. For example, birds living in dense forests have to broadcast songs through more vegetation and over greater distances than woodland birds [15]. These habitat types have higher densities of physical structures that may impede or scatter sound, relative to open habitats, which lack disruptive structures at heights greater than 1 m. We found that the frequency and intensity of sounds were significantly different between the upper and lower parts of the valley in all of the studied species. Our data revealed that in three species, the large-billed crow, common cuckoo, and meadow bunting, the acoustic intensity was significantly different between the upper and lower valley. Compared with the sandy habitat in the upper valley, the virgin forest habitat in the lower valley may affect the sound transmission of the calls of these birds, which may prevent receivers from hearing auditory messages effectively. It may be that topographical factors influence the transmission of the calls of these species, which may prevent receivers from hearing auditory messages effectively in the lower valley; thus, we infer that these species may not survive in the environment of the lower valley.

Animal communication involves a sender producing a signal that travels through an environment and is ultimately detected by a receiver [1]. The properties of sound transmission differ among habitats and may drive the evolution of vocal signals in different directions [34]. For example, bird diversity is highest in primary forests, followed by secondary forests, and artificially planted forests [35]. The transmission of the brown-headed cowbird’s (*Molothrus ater*) song was affected by habitat type [36]. In this study, we also found that birds preferred to live in the upper valley rather than the lower valley, i.e., bird diversity was greater in the upper valley than in the lower valley. This may be due to the effects of habitat structures in the lower valley on the transmission and reception of bird calls.

## 5. Conclusions

The acoustic environment has a major influence on animal sound transmission. Our data on the differences in acoustic parameters may explain that birds will change the frequency and intensity of sound in different habitats. The vocalisations of some birds differ between the two parts of the valley, which may be due to the impact of the environment on the transmission and reception of sound; this may in turn cause birds to leave the lower valley. Further, sound transmission differs among habitats and may promote the evolution of bird sound signals in different directions as a long-term adaptation to different environments. In summary, our study may help to explain the impact of valley topography on avian acoustic communication. We hope that the results and knowledge of this study will benefit the conservation and management of birds in the Daqinggou National Nature Reserve. In addition, the vocalisations of amphibians and mammals that differ between the two parts of the valley may be considered in the future. 

## Figures and Tables

**Table 1:** The differences of fundamental and formant frequencies of the bird sounds at the distance of 30 m between the upper and lower parts of Daqinggou valley.

Variables	Experimental Group		t	df	*p* Value (Two-Tailed)
	Upper Valley (Hz)	Lower Valley (Hz)			
Large-billed crow F0	499.33 ± 0.80 (6)	492.48 ± 3.02 (8)	2.193	7.95	0.060
Common cuckoo F0	919.75 ± 10.97 (5)	942.45 ± 8.67 (5)	−1.623	7.59	0.143
Eurasian magpie F0	2574.33 ± 18.92 (4)	2425.02 ± 3.71 (5)	8.708	7	0.000 *
Meadow bunting F0	3876.19 ± 4655 (5)	4016.96 ± 39.98 (5)	−2.294	8	0.051
Eurasian tree sparrow F0	3346.08 ± 17.52 (5)	3443.77 ± 61.93 (5)	−1.518	4.64	0.194
Large-billed crow F1	1054.41 ± 9.36 (6)	1073.87 ± 4.46 (8)	−2.041	12	0.064
Common cuckoo F1	913.42 ± 13.23 (5)	933.79 ± 11.40 (5)	−1.166	8	0.277
Eurasian magpie F1	2412.57 ± 76.49 (4)	1950.13 ± 14.39 (5)	5.942	3.21	0.008 *
Meadow bunting F1	1421.25 ± 15.53 (5)	1331.42 ± 3.53 (5)	5.639	4.41	0.004 *
Eurasian tree sparrow F1	1254.34 ± 2.91 (5)	1363.32 ± 11.78 (5)	−8.983	8	0.000 *
Large-billed crow F2	2209.87 ± 42.66 (6)	1580.28 ± 4.46 (8)	14.677	5.11	0.000 *
Common cuckoo F2	1912.68 ± 31.30 (5)	1865.65 ± 19.28 (5)	1.279	8	0.237
Eurasian magpie F2	3776.22 ± 52.66 (4)	2813.15 ± 19.98 (5)	18.699	7	0.000 *
Meadow bunting F2	2998.84 ± 8.90 (5)	2874.26 ± 24.03 (5)	4.862	8	0.001 *
Eurasian tree sparrow F2	2847.09 ± 3.03 (5)	2707.08 ± 8.89 (5)	14.910	8	0.000 *
Large-billed crow F3	3091.92 ± 23.33 (6)	2748.11 ± 6.37 (8)	16.17	12	0.000 *
Common cuckoo F3	2992.58 ± 15.15 (5)	2865.22 ± 18.75 (5)	5.284	7.66	0.001 *
Eurasian magpie F3	5497.46 ± 37.02 (4)	4729.90 ± 14.76 (5)	20.991	7	0.000 *
Meadow bunting F3	4212.44 ± 8.09 (5)	4104.25 ± 15.98 (5)	6.040	5.93	0.001 *
Eurasian tree sparrow F3	3933.76 ± 9.63 (5)	3884.11 ± 13.34 (5)	3.017	8	0.017 *

Notes: The variables in the table are the fundamental frequency (F0), the first resonance peak (F1), the second resonance peak (F2), and the third resonance peak (F3). ’Upper’ means that the experimental position was above the valley, and ‘lower’ means that the experimental position was below the valley. The number in parenthesis is the sample size. * indicates a significant difference between the two groups.

**Table 2:** The differences of fundamental and formant frequencies of the bird sounds at the distance of 50 m between the upper and lower parts of Daqinggou valley.

Variables	Experimental Group		t	df	*p* Value (Two-Tailed)
	Upper Valley (Hz)	Lower Valley (Hz)			
Large-billed crow F0	490.85 ± 0.98 (5)	473.04 ± 3.39 (6)	4.525	9	0.001 *
Common cuckoo F0	905.64 ± 3.91 (5)	952.04 ± 2.76 (5)	−9.696	8	0.000 *
Eurasian magpie F0	2258.81 ± 99.68 (3)	2365.54 ± 8.33 (5)	−1.047	2.03	0.404
Meadow bunting F0	4564.04 ± 46.96 (3)	4187.05 ± 47.56 (5)	5.229	6	0.002 *
Eurasian tree sparrow F0	3652.67 ± 73.19 (5)	3417.79 ± 121.74 (5)	1.654	8	0.137
Large-billed crow F1	998.54 ± 8.58 (5)	1100.40 ± 6.78 (6)	−9.627	9	0.000 *
Common cuckoo F1	885.73 ± 8.04 (5)	906.25 ± 4.27 (5)	−2.253	8	0.054 *
Eurasian magpie F1	1852.36 ± 106.87 (3)	2064.65 ± 4.82 (5)	−1.984	2.01	0.185
Meadow bunting F1	1296.77 ± 93.96 (3)	1375.23 ± 31.25 (5)	−0.977	6	0.366
Eurasian tree sparrow F1	1424.76 ± 84.30 (5)	1679.31 ± 41.76 (5)	−2.706	8	0.027 *
Large-billed crow F2	1547.25 ± 10.04 (5)	1632.81 ± 23.84 (6)	−3.824	9	0.004 *
Common cuckoo F2	1911.95 ± 47.00 (5)	1758.90 ± 14.43 (5)	3.113	4.75	0.014 *
Eurasian magpie F2	2924.15 ± 177.11 (3)	2929.80 ± 31.67 (5)	−0.031	2.13	0.978
Meadow bunting F2	2983.28 ± 88.90 (3)	2771.21 ± 18.49 (5)	3.054	6	0.022
Eurasian tree sparrow F2	3265.79 ± 19.76 (5)	2756.42 ± 63.74 (5)	7.632	8	0.000 *
Large-billed crow F3	2917.70 ± 7.06 (5)	2742.13 ± 4.23 (6)	22.199	9	0.000 *
Common cuckoo F3	3035.97 ± 66.63 (5)	2708.90 ± 16.43 (5)	4.766	4.49	0.007 *
Eurasian magpie F3	4808.91 ± 73.69 (3)	4936.86 ± 22.38 (5)	−2.079	6	0.083
Meadow bunting F3	4542.81 ± 70.61 (3)	4094.60 ± 6.78 (5)	6.319	2.04	0.023 *
Eurasian tree sparrow F3	4247.70 ± 8.97 (5)	3890.67 ± 25.40 (5)	13.255	4.98	0.000 *

Notes: The variables in the table are the fundamental frequency (F0), the first resonance peak (F1), the second resonance peak (F2), and the third resonance peak (F3). ’Upper’ means that the experimental position was above the valley, and ‘lower’ means that the experimental position was below the valley. The number in parenthesis is the sample size. * indicates a significant difference between the two groups.

**Table 3:** The difference of acoustic intensities of the bird sounds between the upper and lower parts of Daqinggou valley.

Species	Experimental Group		*t*	df	*p* Value (Two-Tailed)
	Upper Valley (dB)	Lower Valley (dB)			
At the distance of 30 m					
Large-billed crow	64.30 ± 0.31 (6)	74.02 ± 0.23 (8)	−25.491	12	0.000 *
Common cuckoo	61.22 ± 0.64 (5)	63.96 ± 0.34 (5)	−3.778	8	0.005 *
Eurasian magpie	64.10 ± 0.82 (4)	66.69 ± 0.25 (5)	−3.337	7	0.012 *
Meadow bunting	62.63 ± 0.16 (5)	60.33 ± 0.11 (5)	11.510	8	0.000 *
Eurasian tree sparrow	62.80 ± 0.20 (5)	60.00 ± 0.32 (5)	7.435	8	0.000 *
At the distance of 50 m					
Large-billed crow	62.58 ± 0.20 (5)	65.38 ± 0.35 (6)	−6.589	9	0.000 *
Common cuckoo	57.38 ± 0.30 (5)	60.66 ± 0.54 (5)	−5.283	8	0.001 *
Eurasian magpie	52.00 ± 1.39 (3)	59.98 ± 0.30 (5)	−5.613	2.19	0.025 *
Meadow bunting	56.10 ± 0.25 (3)	52.82 ± 0.37 (5)	6.254	6	0.001 *
Eurasian tree sparrow	48.82 ± 0.76 (5)	51.24 ± 0.91 (5)	−2.041	8	0.076

Note: The number in parenthesis is the sample size. * indicates a significant difference between two groups.

**Table 4:** Comparison of bird species diversity indices between the upper and lower parts of Daqinggou valley.

Variables	Experimental Group		*t*	*p* Value (Two-Tailed)
	Upper Valley	Lower Valley		
Species number	17.00 ± 2.16	7.00 ± 1.35	−3.922	0.008 *
Species richness Margalef’s index	3.59 ± 0.33	2.02 ± 0.19	−4.084	0.006 *
Shannon–Wiener index	0.32 ± 0.05	0.52 ± 0.08	2.040	0.087
Pielou’s evenness index	1.31 ± 0.17	1.29 ± 0.04	−0.073	0.944
Simpson’s dominance index	0.88 ± 0.03	0.76 ± 0.05	−2.101	0.080

Note: * indicates a significant difference between two groups.

## Data Availability

The data that support the findings of this study are openly available in any publicly accessible repository, such as Dryad, as soon as this manuscript is accepted.
